# Altered Oscillation and Synchronization of Default-Mode Network Activity in Mild Alzheimer’s Disease Compared to Mild Cognitive Impairment: An Electrophysiological Study

**DOI:** 10.1371/journal.pone.0068792

**Published:** 2013-07-11

**Authors:** Fu-Jung Hsiao, Yuh-Jen Wang, Sui-Hing Yan, Wei-Ta Chen, Yung-Yang Lin

**Affiliations:** 1 Department of Education and Research, Taipei City Hospital, Taipei, Taiwan; 2 Department of Neurology, Taipei City Hospital, Taipei, Taiwan; 3 Institute of Brain Science, School of Medicine, National Yang-Ming University, Taipei, Taiwan; 4 Department of Neurology, School of Medicine, National Yang-Ming University, Taipei, Taiwan; 5 Laboratory of Neurophysiology, Department of Medical Research and Education, Taipei Veterans General Hospital, Taipei, Taiwan; 6 Department of Neurology, Taipei Veterans General Hospital, Taipei, Taiwan; National Yang-Ming University, Taiwan

## Abstract

Some researchers have suggested that the default mode network (DMN) plays an important role in the pathological mechanisms of Alzheimer’s disease (AD). To examine whether the cortical activities in DMN regions show significant difference between mild AD from mild cognitive impairment (MCI), electrophysiological responses were analyzed from 21 mild Alzheimer’s disease (AD) and 21 mild cognitive impairment (MCI) patients during an eyes closed, resting-state condition. The spectral power and functional connectivity of the DMN were estimated using a minimum norm estimate (MNE) combined with fast Fourier transform and imaginary coherence analysis. Our results indicated that source-based EEG maps of resting-state activity showed alterations of cortical spectral power in mild AD when compared to MCI. These alterations are characteristic of attenuated alpha or beta activities in the DMN, as are enhanced delta or theta activities in the medial temporal, inferior parietal, posterior cingulate cortex and precuneus. With regard to altered synchronization in AD, altered functional interconnections were observed as specific connectivity patterns of connection hubs in the precuneus, posterior cingulate cortex, anterior cingulate cortex and medial temporal regions. Moreover, posterior theta and alpha power and altered connectivity in the medial temporal lobe correlated significantly with scores obtained on the Mini-Mental State Examination (MMSE). In conclusion, EEG is a useful tool for investigating the DMN in the brain and differentiating early stage AD and MCI patients. This is a promising finding; however, further large-scale studies are needed.

## Introduction

The default mode network (DMN) in the brain is characterized by consistent activation during a resting-state condition, that is, when not experiencing attention demand or cognitive load, and deactivation while performing a cognitive task [1], [2]. The DMN consists of anatomically distant regions of the brain, including the posterior cingulate cortex (PCC), precuneus (PCu), inferior parietal cortex (IPC), medial temporal (MT) lobes, medial frontal cortex (MFC), and anterior cingulate cortex (ACC) [1], [2], [3]. The DMN has been associated with the recollection of autobiographical information and self-projection in a situation [4], theory of mind and social cognition [5], mind wandering and daydreaming [6], episodic memory [7], emotion and anxiety [8], self-referential processes [9], and low-level attentional focus [10]. Abnormalities of the DMN have been correlated with mental disorders, such as Alzheimer’s disease [11], [12], schizophrenia [13], depression and anxiety [14], epilepsy [15], autism spectrum disorder [16], attention deficit/hyperactivity disorder [17], and various other conditions. Notably, PCu, PCC and the hippocampus were thought to be preferentially vulnerable to atrophy in AD [18]. Global atrophy can affect areas of connectivity between the PCC and lateral parietal regions and has also been associated with the progression of dementia [19]. Therefore, the brain regions of the DMN are suggested to be those that are most sensitive to neurodegenerative processes [20]. Furthermore, activity in DMN can be readily elicited during resting-state conditions, which may counter the detrimental effects of low cognitive ability.

Alzheimer’s disease (AD) is a neurodegenerative disorder that is characterized by cognitive deficits and behavioral disturbances, as well as pronounced insults to the frontal, temporal and parietal neocortical association areas [21]. Because of the disconnection of corticocortical projections observed in AD, AD has been proposed as a disconnection syndrome [22]. Mild cognitive impairment (MCI) is a transitional stage from normal aging to AD, which has been often reported as a decline in memory with the preserved cognitive and functional abilities, as evidenced by neuropsychological examination [23]. Similar to AD, diffuse amyloid deposits in the neocortex and neurofibrillary tangles in the medial temporal lobe have been found in MCI patients [24]. Presently, there is no cure for AD, whereas treatment intervention at the early stage of AD could delay the onset and progress of this disease. Furthermore, there is no efficient method to determine whether MCI would convert into AD.

Recently, because of the putative association between the DMN and working memory [3], [11], [12], cortical activations of the DMN have been investigated to assess the underlying pathology of AD. Studies using fMRI during cognitive [3] or resting-state tasks [12] have reported that PCC and MT (especially in hippocampus) activity or functional connectivity among specific brain regions of the DMN may discriminate AD patients from aging controls. Moreover, MCI participants may be distinguished from AD patients by the magnitude of deactivation in the ACC [25], and MCI patients exhibit diminished resting-state connectivity in the DMN [26]. Though these fMRI studies reported characteristics of DMN activities in AD, some researchers argued against the use of fMRI, as fMRI does not provide direct information from the actual neural elements that underlie all of our cognitive capacities [27], [28]. Moreover, the underlying network patterns coordinating ongoing cognitive functions change much more rapidly (i.e., within fractions of a second) [29], [30]. Therefore, these fMRI studies only examined the slow resting-state oscillations due to the limitation of temporal resolution. Other recording modalities that provide better temporal information and represent neural activation during cognitive tasks are sought. These techniques may increase the understanding of the functional significance of different neurophysiologic mechanisms, in particular, those that underlie the neurodegenerative effects in AD that can influence ongoing mental activities at rest. EEG is a good candidate to study the temporal dynamics of ongoing brain activity in the DMN because it has high temporal resolution and can provide a relevant picture of summated neural activities.

Various studies report that EEG can be used to characterize the manifestation and progression of AD by the increase of delta and theta activities [31], [32], [33] and decreased coherence among brain regions [21], [34], [35], [36], [37], [38]. Previous studies using EEG source-based spectral power analysis suggest that EEG could provide a useful measure to investigate pathology-related cortical abnormalities during rest in MCI and AD [34], [39], [40], [41], [42]. These findings illuminate the need for further investigations exploring the spatial detail and resting-state EEG rhythm synchronization of brain regions of the DMN in MCI and mild AD. The findings from these investigations could potentially elucidate neuropathological changes that underlie the pathologic mechanisms of neurodegeneration.

The present study aimed to measure the oscillatory power and functional connectivity within the DMN via the resting-state EEG activity in AD and MCI subjects. The study hypothesis was that the rhythms of cortical DMN regions show significant difference between mild AD and MCI, and they are correlated with neuropsychological test performance (Mini-Mental State Examination, MMSE).

## Methods

### Subjects and Inclusion/Exclusion Criteria

This study retrospectively enrolled 21 AD patients and 21 MCI patients with scalp EEG examinations. AD subjects were diagnosed according to NINCDS-ADRDA [43] and DSM IV criteria. Amnesic MCI was diagnosed according to previous published guidelines [44]. Each subject was first visit to the neurological institute of Taipei City Hospital during 2007–2011 and complete clinical data regarding dementia or cognitive decline are available including clinical histories, neurological examinations, neuroimaging studies (CT or MRI), neuropsychological interview, MMSE [45] and CDR (clinical dementia rating) [46]. These data were reviewed by an expert neurologist (YJW) to exclude subjects with brain lesions or other abnormalities that can lead to atypical AD symptoms, such as frontotemporal dementia, vascular dementia, extrapyramidal syndromes, reversible dementias, and Lewy body dementia.

The inclusion criteria for AD was CDR = 1. The inclusion criteria for MCI subjects were: (1) CDR score of 0.5; and (2) MMSE score of 20–25. The exclusion criteria for both AD and MCI in this study were (1) no daily use of medications prior to EEG recording; (2) evidence of other neurological or psychiatric diseases characterized by the cognitive impairment; and (3) uncontrolled or complicated systemic diseases or traumatic brain injuries. The neurologist disregarded the reports of EEG examination to avoid sources of bias and confounding in this retrospective investigation. Informed consent of each subject was not collected due to the retrospective design. This study with the waiver of informed consent was approved by the Institutional Review Board of Taipei City Hospital.

Table 1 summarizes the demographic and neuropsychological data collected from both groups. No significant age differences were observed between the AD and MCI groups. A slight, but non-significant decrease in individual alpha frequency was discovered in the AD group compared to the MCI group. MMSE scores were significantly lower in the AD group than in the MCI group (p<0.0001); the scores of both groups were below normal (MMSE cutoff value >25/30).

**Table 1:** Demographic information of AD and MCI subjects.

	AD (n = 21)	MCI (n = 21)	
Gender	6M/15F	8M/13F	
Age	82.7±7.5	80.4±5.6	P>0.05
IAF	8.7±0.6	9.1±0.7	P>0.05
CDR	1	0.5	
MMSE	14.9±5.4	22.7±2.2	P<0.0001

IAF, individual alpha frequency; CDR, clinical dementia rating; MMSE, mini-mental state estimate.

### EEG Recordings and Preprocessing

All EEG data were recorded using a Nihon-Kohden system (Nihon-Kohden Inc., Tokyo, Japan) with 19 electrodes, which were positioned according to the International 10–20 System and consisted of Fp1, Fp2, F3, F4, C3, C4, P3, P4, O1, O2, F7, F8, T3, T4, T5, T6, Fz, Cz, and Pz. The EEG activities were commonly referenced to the average of the two linked mastoid electrodes, sampled at 200 Hz, and filtered offline between 1 and 40 Hz. Both eyes-closed and eyes-opened conditions were recorded for 20–30 s alternately to a total of 3 minutes for each condition. Subjects remained awake and alert during the recording without increased attentional demand or cognitive load. The EEG data of the eyes-closed condition were extracted and fragmented into consecutive epochs of 2 s in off-line mode, whereas the data during activation procedures of the routine clinical EEG recording such as hyperventilation and photic stimulation were discarded in the present study. For each subject, 30 artifact-free epochs were randomly selected for further analysis.

To eliminate interference caused by ocular, muscular, heart and other types of artifacts, two processes were used. First, the data were reviewed, and the epochs with aberrant waveforms were manually discarded by an expert EEG technician. Second, detection and rejection of artifacts were completed using the independent component analysis (ICA) of the EEGLAB toolbox (available at sccn.ucsd.edu/eeglab); this procedure was extremely effective in decomposing the signals into multiple statistically independent components, allowing artifacts to be easily detected [47]. The components were visually inspected and, if artifact contamination, manually rejected by the investigator (FJH). The rejection criteria were that: (1) the topographic voltage map was far-frontal projection with smoothly decreasing EEG spectrum which is typical of eye artifacts, or (2) the map was marginally localized and showed high power at high frequencies (20–50 Hz or above). The number of rejected components was not significantly different between MCI and AD patients (MCI, 3.9±0.7; AD, 4.1±0.8; t = −0.803, p = 0.427). By the ICA denoising procedure, the rejected components with marginal locations and specific spectra, which are distinct from the cortical activities of DMN, would slightly distort the EEG activities in DMN. Moreover, prior studies have shown that ICA performs effectively in attenuating artifacts to obtain clear EEG signals [48], [49].

### Data Analysis

#### (1) Minimum norm estimate analysis

Depth-weighted minimum-norm estimation (MNE) was used to obtain the current strength dynamics of cortical sources of the EEG data [50]. This method offers greater spatial accuracy than MNE without depth weighting [51] and is able to detect simultaneous current sources that are distributed along the entire cortical surface [50]. In this study, the electrode locations onto the scalp were based on the default coordinates. The forward model used a symmetric boundary element method (symmetric BEM), which was developed by the French public research institute INRIA (http://www-sop.inria.fr/athena/software/OpenMEEG/). To compute the inverse operator, (a) the source orientations were constrained to be normal to the cortical surface; (b) a depth weighting algorithm was used to compensate for the biased calculations of superficial sources [52]; and (c) a regularization parameter, *λ* ^2^ = 0.33, was used to minimize numerical instability, reduce the sensitivity to noise of the MNE, and effectively obtain a spatially smoothed solution [50]. In the present analysis, the activation at each vertex (an equilateral triangle in the tessellation of the cortical surface) was estimated every 5 ms. Cortical maps of distributed current activity in each subject were displayed on the same source space: the cortex from Colin27 anatomy. The regions of interest (ROIs), which included the PCC, PCu, IPC, MT, MFC, and ACC in the bilateral hemispheres, were selected from the cortical surface of default anatomy (MNI/Colin27) according to the automatic anatomical labeling template [53]. The time-varying current strengths at these ROIs were extracted in each epoch, and averaged cortical activation across time and epochs was calculated for each ROI in each subject. MNE analysis was performed with Brainstorm [54], which is a documented program that is available for free download online under the GNU general public license (http://neuroimage.usc.edu/brainstorm).

#### (2) Spectral power dynamic and functional connectivity analysis

To obtain the source-based spectral power of activity in the DMN, FFT spectral analysis was used to transform the time-varying current strength of each ROI, in each epoch, into power spectrums of 0.39 Hz frequency resolution. All spectrums were further averaged across epochs. Because of the inter-individual differences, the frequency ranges of EEG rhythms should differ from subject to subject [55]. The individual alpha frequency (IAF) peak was determined before making an oscillatory power estimation. The difference of IAF between mild AD and MCI was not significant (Table 1). The frequency bands in this study were selected and classified into delta (IAF-8 to IAF-6 Hz), theta (IAF-6 to IAF-2 Hz), alpha1 (IAF-2 to IAF Hz), and alpha2 (IAF to IAF+2 Hz). Beta1 (13–20 Hz), beta2 (20–30 Hz) and gamma (30–40 Hz) bands were also considered. The spectral power was normalized by means of dividing the power at each frequency band by the total power from delta to gamma, which has been reported to adequately reduce the inter-individual variability in previous EEG studies [34].

Imaginary coherence (IC) was used to estimate the functional connectivity for the capability to minimize the crosstalk effects between sources due to the spatial resolution of EEG inverse modeling [56]. In addition, this technique has been suggested to effectively index deficits related to functional connectivity in brain lesions [57] and brain tumors [58]. Based on evidence suggesting that there is no time delay between two distinct cortical activities of a common source or volume conduction, actual components of coherence, which mostly has no time delay, may be discarded. Thus, imaginary coherence could represent the interactions between brain regions with a specific time lag [56]. IC was calculated according to the following formula:
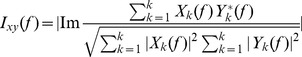
where *I_xy_(f)* is the IC between a given paired-ROIs for each frequency bin, *Im* is the imaginary part of the complex production, *X_k_* and *Y_k_* are the source-based spectrums from two ROIs, * denotes the complex conjugate, and *K* is the number of 2s epochs.

The IC values for each frequency band were calculated from the average of all epochs. Fisher’s z-transformed IC values were used to obtain the grand averaged values over subjects [56], by the following formula:
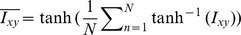
where N indicates the number of subjects.

### Statistical Analysis

An ANOVA was performed to examine significant differences for the effect of Group (mild AD and MCI) and Region (ROIs) on the current strength of cortical sources. Source-based normalized spectral powers were tested using an ANOVA with the factors Group (mild AD and MCI), Band (delta, theta, alpha1, alpha2, beta1, beta2 and gamma) and Region (12 ROIs). The Bonferroni correction for multiple comparisons was used as post-hoc analysis. To identify the difference of IC values between 2 groups with respect to 12 ROIs and 7 frequency bands, the False Discovery Rate (FDR) was used to correct for multiple comparisons (66×7 = 462) [56]. FDR can control for the expected proportion of incorrectly rejected null hypotheses and is a less conservative procedure than the Bonferroni correction [59]. Additionally, Pearson’s correlation analysis was used to calculate the correlation between (1) the normalized oscillatory powers and MMSE scores and between (2) the IC values and MMSE scores.

Statistical analysis was carried out using Matlab toolbox (version 7.10, R2010b, http://www.mathworks.com/) or SPSS software package (SPSS Inc, Chicago). A p-value <0.05 was considered statistically significant.

## Results

### Cortical Activation Analysis

**Figure 1:**
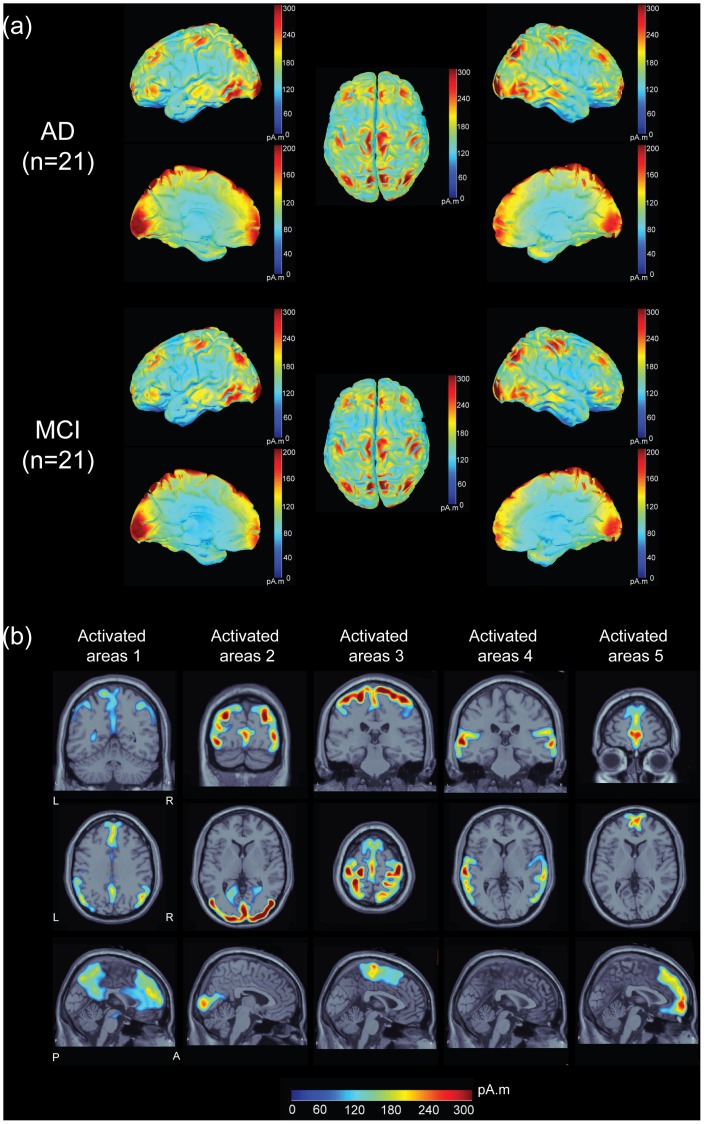
Resting state networks from EEG data. (a) Distributions of averaged cortical activation during an eyes-closed resting-state condition in 21 AD and 21 MCI patients, respectively. (b) 5 activated maps on the sagittal, coronal and axial MR images are shown. The sources values were smoothed after being re-interpolated (the size of the smoothing kernel = 5). The current strength of cortical sources is color coded; large values are represented in red, and small values are in blue.

Fig. 1(a) illustrates the grand-averaged activation distributions on cortical surfaces at all frequency bands with lateral, medial and dorsal views across the AD and MCI patients, respectively. The activation maps distributing over the frontal, parietal, temporal, occipital and midline regions were similar between two groups. The current strength of underlying cortical sources is color coded. Locations of activated brain regions from AD patients were mapped onto the axial, coronal, and sagittal MR images (Fig. 1(b)). Five activated areas were identified from the major foci of activation maps: DMN regions (area 1), posterior occipital and temporal-occipital regions (area 2), sensory-motor cortices and supplementary motor area (area 3), bilateral auditory and lateral temporal cortices (area 4), and medial-ventral prefrontal cortex (area 5). Brain areas are colored in blue-red denoted the small-large activation.

**Figure 2:**
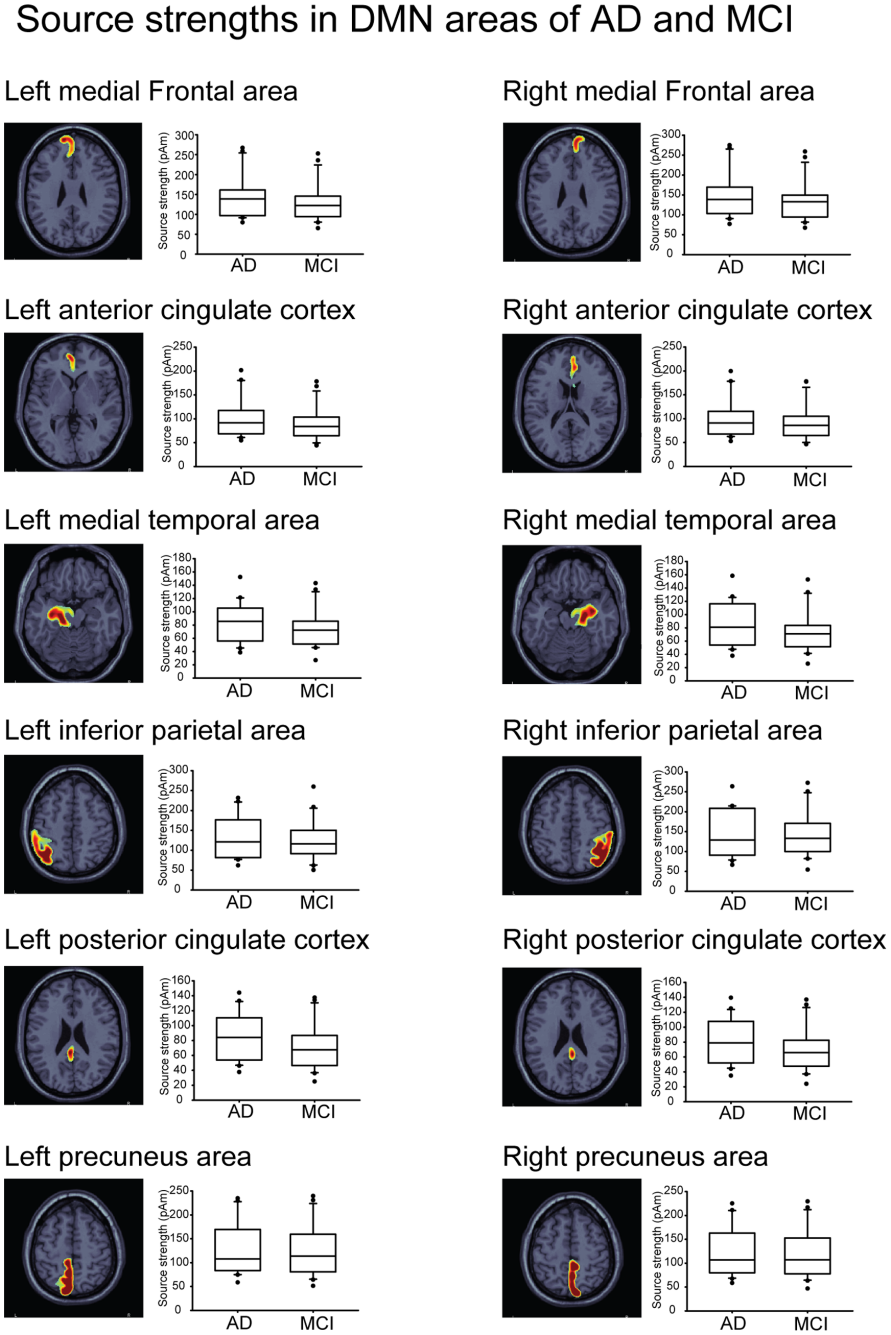
Box plots of averaged current strength of 12 ROIs in AD and MCI.

Fig. 2 shows the box plots of the cortical activation of each ROI within the DMN in AD and MCI with respect to the degree of dispersion, skewness and outliers. The box plots display the distribution of data based on the five numbers: minimum, first quartile, median, third quartile, and maximum. A segment inside the rectangle shows the median and whiskers above and below the box show the locations of the minimum and maximum. The outliers are surprisingly high maximums or low minimums. For the current strength of cortical activity, we found a main effect for Region (F(11,440) = 15, p<0.0001) and Group (F(1,40) = 5.48, p<0.05); however, there was no significant Group×Region interaction (F(11,440) = 0.05, p>0.05). Post-hoc tests for the factor Region revealed that the current strength was greater in the bilateral MFC than in the ACC (p<0.005), PCC (p<0.001) and MT (p<0.001); greater in the bilateral PCu than in the PCC (p<0.001) and MT (p<0.005); and greater in the bilateral IP than in the ACC (p<0.05), PCC (p<0.001) and MT (p<0.001). Within-groups comparisons showed that the mean difference of current strength in AD and MCI was about 10 pAm (AD, 113.2 pAm; MCI, 103.8 pAm). There was no main interaction effect between the two factors; similar current strength was found in each ROI between the AD and MCI groups.

### Spectral Power Analysis

Statistical examination of the normalized spectral power values yielded a significant main effect for Band (F(6,240) = 1080.7, p<0.00001) and Region (F(11,440) = 2.16, p<0.05). Post-hoc analysis of Band yielded significantly different values in the following descending order: alpha1> theta>delta>alpha2> beta1> beta2> gamma, with all p-values <0.0001. For the effect of Region, the bilateral PCC were larger than the bilateral MFC (p<0.05) and ACC (p<0.05); furthermore, the bilateral PCu, MFC and ACC were larger than the left MFC and left MT (all p<0.05).

**Figure 3:**
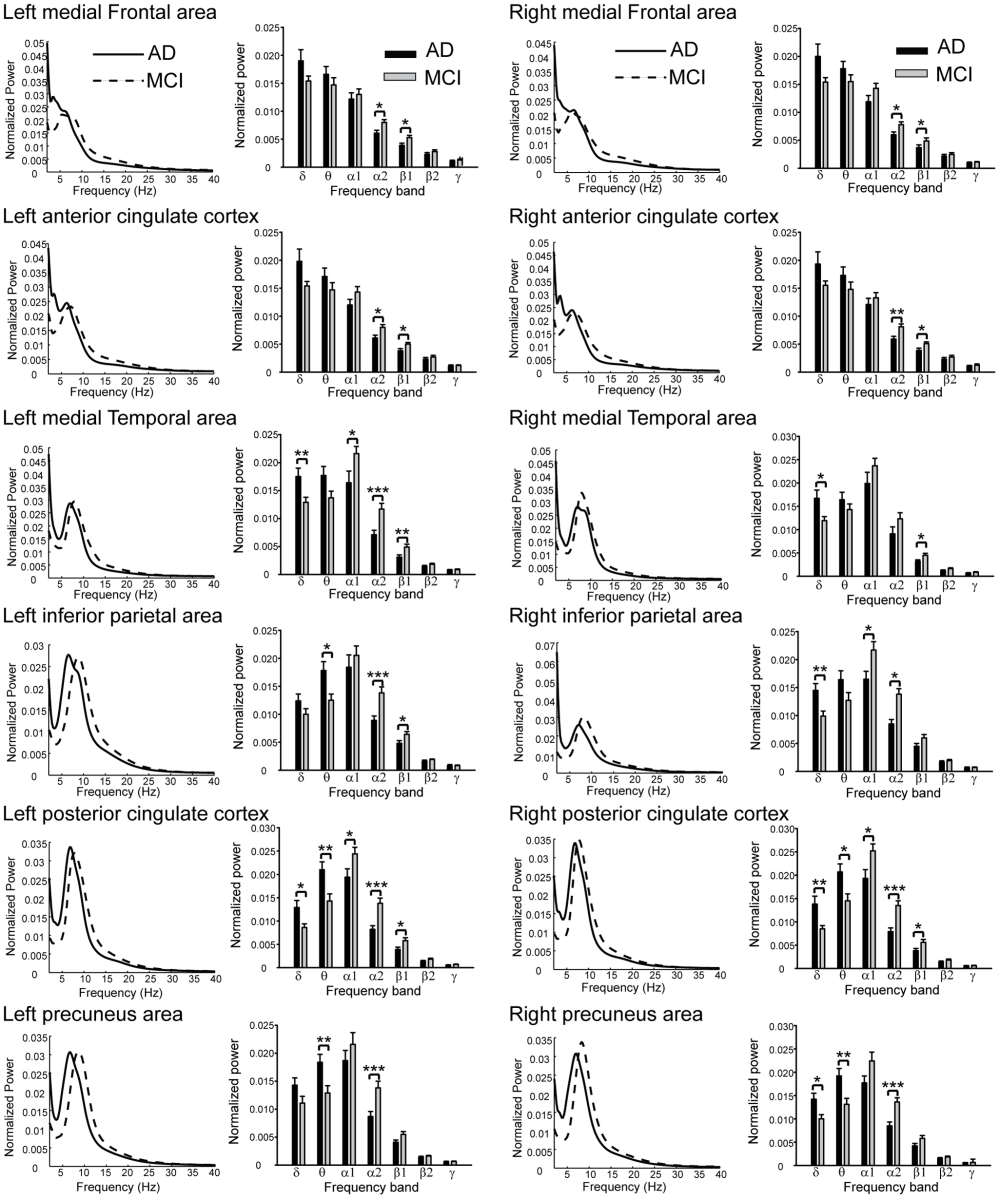
The estimated normalized and averaged power spectrum of 12 ROIs at 1–40 Hz across AD and MCI, respectively. The bar plots represent the normalized power values in each frequency band in AD and MCI. δ, delta; θ, theta; α1, alpha1; α2, alpha2; β1, beta1; β2, beta2; γ, gamma. *, p<0.05; **, p<0.01; ***, p<0.001.

The ANOVA yielded a significant interaction between the factors Group, Band, and Region, as shown in Fig. 3. The averaged spectrum from each ROI in the AD and MCI groups was ranged in frequency from 1 to 40 Hz. With regard to the larger power in the MCI group compared to the AD group, alpha2 was found in all ROIs except for the right MT. On the other hand, beta1 was larger in the bilateral MFC, ACC, MT and PCC, and left IP. Furthermore, the difference between groups was noted in the alpha1 band in the left MT, right IP and bilateral PCC. As for the smaller power in MCI compared to AD, delta activity was observed in the right IP and PCu, and bilateral MT and PCC, and theta activity was observed in the left IP and bilateral PCC and PCu. In summary, the spectral pattern of cortical sources of the DMN exhibited attenuation in alpha and beta bands and augmentation in delta and theta bands in AD. Regarding the spectral peak frequency, the ANOVA also found a significant effect for the factor Group. The peak frequencies in the AD were smaller than those in the MCI, significantly in the bilateral PCC (left, p = 0.001; right, p<0.0001), PCu (left, p = 0.025; right, p<0.0001) and MT (left, p = 0.005; right, p = 0.012), and right IP (p<0.0001).

Table 2 lists the correlation coefficients and their significance levels between MMSE scores and normalized spectral power values. Positive correlations were found in alpha1 and alpha2 bands in the bilateral IP, PCC and PCu. In the bilateral MT, the power values in the alpha1 band were also positively correlated with MMSE scores. Negative correlations were found in the left MT and bilateral IP, PCC and PCu in the theta band. In the delta band, a similar relationship was detected in the right IP and PCu.

**Table 2:** Correlation between MMSE and normalized spectral power in the brain regions of DMN.

ROI	Band						
	Delta	Theta	Alpha1	Alpha2	Beta1	Beta2	Gamma
MFC_L	r = −0.072	r = −0.235	r = 0.113	r = 0.265	r = 0.148	r = 0.039	r = 0.223
	p = 0.652	p = 0.134	p = 0.474	p = 0.09	p = 0.349	p = 0.807	p = 0.155
MFC_R	r = −0.006	r = −0.246	r = 0.257	r = 0.288	r = 0.17	r = −0.071	r = −0.032
	p = 0.97	p = 0.116	p = 0.101	p = 0.064	p = 0.281	p = 0.655	p = 0.841
ACC_L	r = 0.021	r = −0.186	r = 0.138	r = 0.188	r = 0.098	r = −0.02	r = 0.163
	p = 0.894	p = 0.24	p = 0.382	p = 0.232	p = 0.536	p = 0.899	p = 0.302
ACC_R	r = −0.04	r = −0.169	r = 0.123	r = 0.279	r = 0.136	r = −0.038	r = 0.109
	p = 0.982	p = 0.284	p = 0.439	p = 0.074	p = 0.391	p = 0.81	p = 0.493
MT_L	r = −0.218	**r = **−**0.33**	**r = 0.342**	r = 0.285	r = 0.233	r = 0.041	r = 0.048
	p = 0.166	**p = 0.033** *	**p = 0.027** *	p = 0.067	p = 0.137	p = 0.794	p = 0.762
MT_R	r = −0.249	r = −0.24	**r = 0.303**	r = 0.091	r = 0.181	r = 0.078	r = 0.125
	p = 0.112	p = 0.126	**p = 0.05** *	p = 0.568	p = 0.251	p = 0.625	p = 0.428
IPC_L	r = 0.22	**r = **−**0.374**	**r = 0.328**	**r = 0.32**	r = 0.084	r = −0.038	r = −0.09
	p = 0.162	**p = 0.015** *	**p = 0.034** *	**p = 0.039** *	p = 0.597	p = 0.813	p = 0.571
IPC_R	**r = **−**0.328**	**r = **−**0.337**	**r = 0.425**	**r = 0.334**	r = 0.119	r = 0.036	r = 0.042
	**p = 0.034** *	**p = 0.029** *	**p = 0.005** **	**p = 0.031** *	p = 0.453	p = 0.819	p = 0.791
PCC_L	r = −0.206	**r = **−**0.379**	**r = 0.377**	**r = 0.345**	R = 0.166	r = 0.047	r = 0.087
	p = 0.19	**p = 0.013** *	**p = 0.014** *	**p = 0.025** *	P = 0.294	p = 0.766	p = 0.582
PCC_R	r = −0.187	**r = **−**0.362**	**r = 0.392**	**r = 0.34**	r = 0.168	r = 0.022	r = 0.015
	p = 0.235	**p = 0.018** *	**p = 0.01** **	**p = 0.028** *	p = 0.288	p = 0.892	p = 0.925
PCu_L	r = −0.254	**r = **−**0.385**	**r = 0.414**	**r = 0.332**	r = 0.138	r = 0.072	r = 0.039
	p = 0.105	**p = 0.012** *	**p = 0.006** **	**p = 0.032** *	p = 0.384	p = 0.651	p = 0.805
PCu_R	**r = **−**0.305**	**r = **−**0.435**	**r = 0.511**	**r = 0.408**	r = 0.141	r = −0.007	r = −0.009
	**p = 0.05** *	**p = 0.004** **	**p = 0.001** **	**p = 0.007** **	p = 0.373	p = 0.967	p = 0.953

ROI, region of interest; L, left hemisphere; R, right hemisphere; MFC, media frontal cortex; ACC, anterior cingulate cortex; MT, medial temporal; IP, inferior parietal cortex; PCC, posterior cingulate cortex; PCu, precuneus;

* p<0.05;

** p<0.01.

### Functional Connectivity Analysis

**Figure 4:**
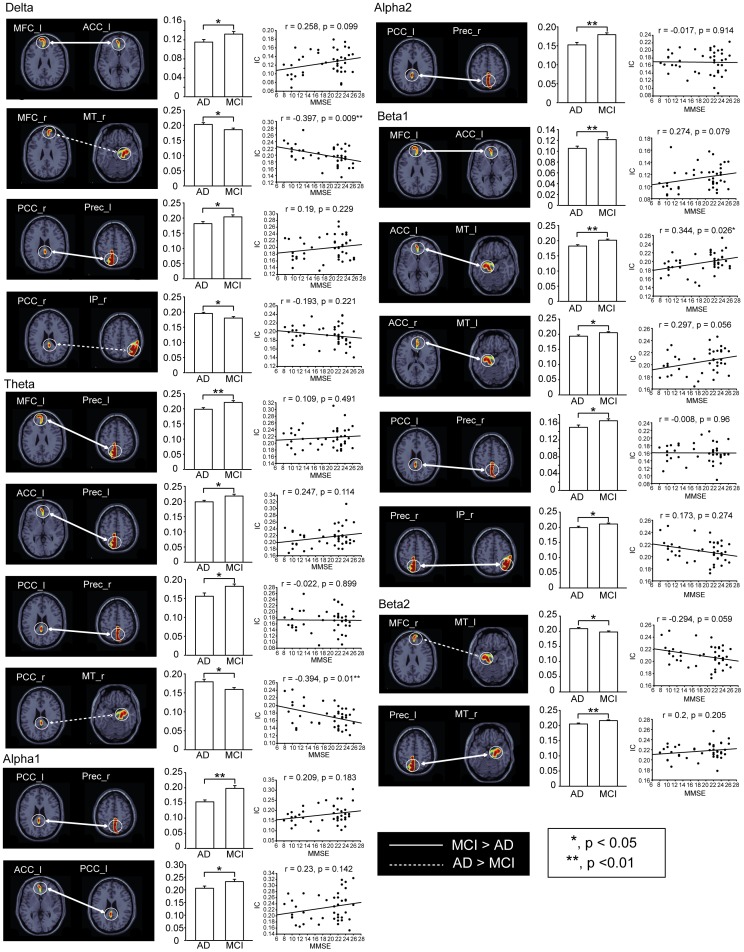
Significant differences of functional connectivity within the DMN with respect to different frequency bands between AD and MCI. The solid line indicates larger IC values between brain regions in MCI, and the dotted line exhibits larger ones in AD. Scatter plots with linear regression represent the correlation between MMSE scores and IC values. *, p<0.05; **, p<0.01; ***.

Fig. 4 shows the corticocortical connections within the DMN, reflecting significant differences between AD and MCI. Enhanced IC values were observed in AD, mainly between the MT and MFC (in the delta band, p<0.05) and the MT and PCC (in the theta band, p<0.05) of the right hemisphere, as well as an interaction between the left MT and right MFC in the beta2 band (p<0.05). Delta IC between the right PCC and IP was greater in AD compared to MCI (p<0.05). In contrast, attenuated functional connectivity was observed in AD. This phenomenon was found in the left PCu, in particular where it connects to the left right PCC (in the delta band, p<0.05), left MFC (in the theta band, p<0.01), left ACC (in the theta band, p<0.05) and right MT (in the beta2 band, p<0.01). This attenuated functional connectivity was also found in the right PCu, in regions interacting with the left PCC (in the theta, alpha1, alpha2 and beta1 bands, all p<0.05) and right IP (in the beta1 band, p<0.05). In addition, deteriorated functional interactions were found in the connections between the left ACC and the left MFC (in the delta and beta1 bands, p<0.05), left PCC (in the alpha1 band, p<0.05) and left MT (in the beta1 band, p<0.01). Similar deterioration was found in connections between the right ACC and the left MT (in the beta1 band, p<0.05).

The scatter plots in Fig. 4 represent the correlation between MMSE scores and IC values. The IC values between the right MT and right MFC (in the delta band) and between the right MT and right PCC (in the theta band) were negatively correlated with neuropsychological performance (all p<0.01). A positive correlation was observed between MMSE scores and IC values, which reflected the alpha2 connectivity between the left MT and left ACC (p<0.05).

## Discussion

This study investigated the cortical activities in the DMN regions and their relationship to functional measures based on the previous findings that these regions are significantly affected by both MCI and AD, showing cellular and structural abnormalities. The present study shows that cortical activities of resting-state oscillations are altered during the transition from MCI to AD. Such alteration is characteristic of attenuated alpha or beta activity in the DMN as well as enhanced delta or theta band activity in the MT, IP, PCC and PCu. In addition, altered synchronization was demonstrated in AD. The connectivity between the PCu, PCC and ACC is disrupted, whereas the MT shows increased connectivity with the MFC and PCC in the delta, theta and beta2 bands. MMSE scores were strongly correlated with the magnitudes of spectral power at the theta and alpha bands in posterior portion of the DMN. Furthermore, the correlations between MMSE scores and the functional connectivity in the DMN were characterized by (i) delta IC between the right MFC and right MT, (ii) theta IC between the right PCC and right MT, and (iii) beta1 IC between the left ACC and left MT.

### Oscillatory Characteristics of Resting EEG

Cortical activity in the DMN were correlated with EEG power and characterized by EEG rhythms of different frequency bands [60], [61]. Regarding abnormal resting-state EEG rhythms, AD patients exhibited a power increase of widespread delta and theta activity as well as a power decrease in posterior alpha and beta activity, as evidenced by electrode-based [31], [32], [33] and source-based spectral analysis [39], [40], [41], [42]. Babiloni and colleagues [39], [40], [41], [42] have elucidated spectral changes of cortical sources in the delta, theta and alpha bands that occur in the frontal, central, parietal, temporal, occipital and limbic regions of AD patients. They also have validated the relationship between the change of cortical rhythms and the severity of AD. The present findings suggest that AD not only weakens the alpha and beta activity of the DMN, which may be responsible for volumetric change and functional abnormality [11], [26], but also the enhancement of delta and theta rhythm reflects the brain activity deficits or cognitive decline [31], [32], [33]. Moreover, the altered oscillations in the DMN indicate the functional and structural changes in AD and agree with prior fMRI studies [3], [12], [26], [62], [63], [64], [65], which suggested that the DMN characterized the neuropathological changes in AD.

### Functional Connectivity within the DMN

Functional deterioration of the DMN in mild AD involves the MT and PCC/PCu [3], [26], [62], [64], [65] and, consequently, disrupts the functional connectivity between these regions and others within the DMN [3], [26], [62], [64], [65]. Previous electrophysiological studies have reported disrupted functional connectivity in AD as well as fewer connections between fronto-parietal and fronto-temporal regions in the alpha and beta frequency bands [34], [35], [36], [37], [38]. The topographic distributions of these electrophysiological changes correlated with regions of the DMN [60], [61]. Our investigation identified potential hubs of disrupted brain connectivity in AD, which support previous studies [3], [62], [65]: the PCu, PCC and ACC. Disrupted connectivity supports the hypothesis that AD is a disconnection syndrome [22]. Furthermore, greater connectivity in AD has been previously reported in EEG/MEG studies in delta and theta bands [37], as well as in beta and gamma bands [37]. Similarly, greater connectivity has been reported in fMRI studies of AD [12], [63], [64]. Enhanced connectivity supports a compensatory hypothesis, which states that patients recruit additional neural resources to maintain cognitive function [12], [63], [64]. Our results suggest the altered functional connectivity within DMN could enrich the characterization of cortical abnormality in the early stage of disease progression in AD.

FDR instead of Bonferroni method was used for the correction of multiple tests in this study. Bonferroni correction assumes that the tests are independent of each other. While the Bonferroni correction is good at controlling the familywise error rate for multiple and independent comparisons, it may conduce a very high rate of false negatives [66]. Notably, the Bonferroni approach does not account for the correlations of IC values among ROI pairs. On the other hand, FDR method generally provides more powerful statistical power than Bonferroni correction but also less certainty regarding the reliability of single statistical test [67]. FDR is also appropriate to limited statistical power (small sample sizes or weak effects) or the decrease of miss effects [68]. To sum up, in order to negotiate the trade-off between the Type I and II errors, as well as the power and the degree of certainty, FDR method might be well suited for present study.

### Correlations between Cortical Activity and the MMSE

MMSE scores were correlated with EEG spectral power [42], [69] and PET glucose metabolism [70] during active task and resting-state conditions. The spectral power at the delta, theta and alpha bands was an indicator of the degree of cognitive decline observed in AD [42], [69]. Notably, theta and alpha oscillations have been suggested to play an important role in cognitive and memory processing [71]. Our results demonstrate that delta, theta and alpha activity are engaged in cognitive performance during rest. Furthermore, previous evidence has indicated that AD severity affects posterior cortical regions such as the posterior cingulate cortex and parietal lobes [70]. Of note, the PCC and PCu are associated with the earliest signs of AD-related pathology, which are closely related to global measures of cognitive decline [26]. The present study demonstrates a potent relationship between cognitive functions in MCI and AD patients and the spectral activities with respect to theta and alpha bands in the IP, PCC and PCu regions of DMN.

Functional connectivity was also associated with the MMSE score, as evidenced by grand-averaged beta band synchronization [72], interhemispheric alpha band synchronization [37], and mean synchronization in alpha and beta bands [35], [36]. Previous fMRI studies have reported reduced connectivity in the hippocampus and posterior parietal regions in AD [73], [74]; this reduced connectivity is a significant predictor of disease progression that is independent of global atrophy [75]. Therefore, the medial temporal lobe is integral to episodic memory processing [76], and altering its connections to other brain regions in the DMN may account for cognitive decline in AD [3], [26], [62], [65], [74]. The present findings indicate that the functional connectivity within the DMN regions, especially those pertinent to the MT region, was strongly correlated with the MMSE score in different frequency bands.

### Methodological Remarks

The weighted MNE method for source reconstruction is known as non-adaptive spatial filters [77]. Sekihara and colleagues have shown no localization bias by using minimum-norm-based method [77]. The improvement of localization accuracy in MNE method was reported by the use of depth weighting [51]. With optimal depth weighting, regularization parameter and noise covariance, MNE exhibited well performance on the localizations of resting state network in the present study (please see Fig. 1). It has been applied independently and successfully in hundreds of studies over the past years, as listed on Brainstorm’s publication pages (http://neuroimage.usc.edu/brainstorm/Pub). Hämäläinen et al. (2010) suggested that MNE and standardized low-resolution electromagnetic tomography (sLORETA) methods should be used in combination to delineate the areas of activation with high signal-to-noise ratio from the significance map and, subsequently, obtain the true current amplitudes at that particular region [78].

### Limitations

The experimental procedure used in the current study appears particularly promising to clarify the differences that exist between MCI and AD in altered spectral power and functional connectivity of the DMN. In order to eliminate the medication effect on the cortical activities, the patients were carefully screened using strict criteria and, therefore small sample size was included. Further studies are needed to confirm these results and extend this investigation to include larger populations. In addition, comparisons between groups of varying severity of cognitive impairment and longitudinal follow-up studies probing into the effect of progression in MCI patients (whether MCI patients have converted to AD or not) may advance the understanding of dementing processes.

### Conclusion

Neurodegeneration and cognitive impairment in AD alter the EEG source-based spectral power and functional connectivity within the DMN. These changes are correlated with clinical measures of cognitive ability. DMN might be useful in assessing the functional change and progression in those at risk for AD. Our findings also demonstrate the need to further validate the clinical relevance of the present approach through longitudinal studies and to test the applicability of DMN connectivity as a clinical predictor of the progression of MCI to AD.
